# Eating Behavior Disorders and Disordered Eating Habits in Spanish High-Performance Women’s Olympic Wrestling Athletes

**DOI:** 10.3390/nu16050709

**Published:** 2024-02-29

**Authors:** Marina Rueda Flores, Adrián Martín-Castellanos, Olga López-Torres, Valentín E. Fernández-Elías, Jorge García-González, Daniel Mon-López

**Affiliations:** 1 Facultad de Ciencias de la Actividad Física y del Deporte (INEF-Departamento de Deportes), Universidad Politécnica de Madrid, C/Martín Fierro, 7, 28040 Madrid, Spain; marina.rueda.flores@alumnos.upm.es (M.R.F.); daniel.mon@upm.es (D.M.-L.); 2 Department of Physical Activity and Sports Science, Alfonso X El Sabio University (UAX), 28691 Madrid, Spain; adrimaca@uax.es; 3 Faculty of Sport Sciences, Universidad Europea de Madrid, 28670 Madrid, Spain; olga.lopez@universidadeuropea.es (O.L.-T.); valentin.fernandez@universidadeuropea.es (V.E.F.-E.); 4 Departamento de Ciencias Sociales de la Actividad Física, del Deporte y del Ocio, Facultad de Ciencias de la Actividad Física y del Deporte—INEF, Universidad Politécnica de Madrid, C/Martín Fierro, 7, 28040 Madrid, Spain

**Keywords:** female wrestling, eating disorder, nutritional habits, sports nutrition

## Abstract

Eating disorders (EDs) are a significant health issue in combat sports. This study investigated the differences between the different types of female wrestlers and the frequency at which EDs occur in the elite population, and it also sought to establish which factors are predictors of EDs. This study was comprised of 22 elite, female wrestlers who were selected based on the following inclusion criteria: having previously been the Spanish champion, being part of the Spanish national team, participating in at least one international championship, and having a history of ED. Data collection involved five questionnaires: demographic data, the Eating Attitudes Test-26 (EAT-26), the Bulimic Investigatory Test, the Edinburgh (BITE), the Eating Disorders Inventory (EDI-3), and the Depression, Anxiety, and Stress Scale (DASS-21). The results revealed diverse levels of depression, anxiety, and stress, with BITE scores indicating abnormal eating patterns. Group comparisons exposed significant distinctions in eating behaviors based on competition and training experience. Regression analyses showed competition and training experience as predictors of bulimia severity and symptoms. The study revealed prevalent extreme weight-control practices, including fasting, diuretic and laxative use, and binge eating. This research emphasizes the importance of EDs in Olympic wrestling, urging a comprehensive approach involving education, support, and policy implementation by coaches, health professionals, and sports organizations to prioritize athletes’ well-being and discourage unhealthy weight-control practices.

## 1. Introduction

Olympic wrestling, which dates back to 708 BC «International Olympic Committee» [1], requires a unique blend of strength, endurance, agility, and technical skill. Wrestlers are classified by body mass to ensure a fair competition in terms of size, strength, and agility [2]; however, as with many weight-controlled sports, concerns about eating disorders (EDs) loom large, as athletes often undergo drastic measures to gain a competitive edge by competing in lighter weight classes [3,4].

Despite the documented health risks associated with rapid weight loss, aggressive weight-loss methods are prevalent in combat sports, particularly in Olympic wrestling [5]. Wrestlers resort to extreme practices such as severe dietary restrictions and deliberate dehydration to meet the weight requirements prior to competition [6]. In addition, weight-category sports have been identified as susceptible to EDs [7].

Clinical EDs include anorexia nervosa, bulimia nervosa, binge eating disorder, and other specified and unspecified EDs [8] and require medical and psychological interventions [9]. While both genders are affected, female athletes are at a higher risk [10], although gender parity in ED cases is increasing nowadays [5].

Awareness of the risks of EDs among coaches, health professionals, and wrestlers themselves is critical [11]. Stress, anxiety, depression, and body-image issues are notable concerns for combat athletes, especially for females [12,13]. These factors often predict EDs and unhealthy eating behaviors, which are enhanced by body dissatisfaction and low self-esteem [14,15], resulting from pressure to maintain weight and meet performance expectations [16].

In addition, chronic stress impairs the immune system and increases the risk of physical injury [17], which can result in difficult recoveries and can impair the performance of Olympic wrestlers under intense competitive pressure [18]. Moreover, anxiety manifests as nervousness, excessive worry, and panic attacks, which can also impair performance and recovery [19,20]. These psychological factors, along with the pressure to maintain weight and meet performance expectations, could increase the risk of EDs among wrestlers.

Furthermore, depression decreases motivation, interest in the sport, and adherence to training regimens, which can put athletic performance at risk [21,22]. Age, experience, and weight control significantly influence the development of EDs; thus, younger, less-experienced athletes face weight-category pressures and lack nutritional knowledge [23], while experienced athletes may adopt unhealthy practices that promote dysfunctional eating patterns [24,25]. 

Thus, addressing these concerns comprehensively through medical, psychological, and emotional support, as well as stress management education, is imperative [11]. Specific research focusing on these issues in female Olympic wrestlers seems to be critical. Hence, the objective of this study was to determine the differences between different categories of female wrestlers, including weight, age, and experience, among others, and the frequencies in which EDs occur in the elite population. In addition, it sought to establish which factors were predictors of EDs.

## 2. Materials and Methods

### 2.1. Participants

**Figure 1:**
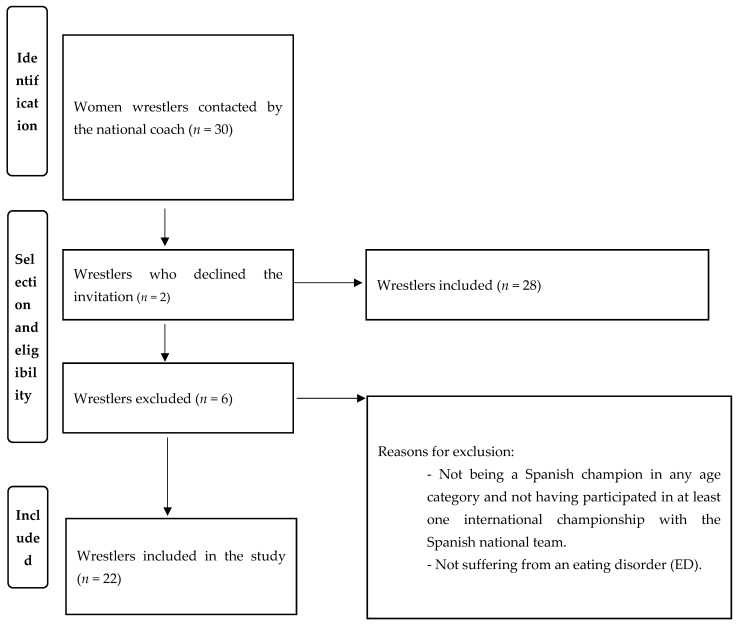
Recruitment process.

Thirty elite, female, Spanish wrestlers were contacted through the national coach. Of the total number of athletes, two declined the invitation and six wrestlers were discarded in accordance with the inclusion/exclusion criteria. The two following inclusion criteria were used [26] to identify subjects who could provide relevant information: (1) elite wrestlers who have been the champion of Spain in any age category and have been part of the Spanish national team, having participated in at least one international championship, and (2) wrestlers suffering from or having suffered from EDs. The final sample was 22 female wrestlers with a mean age of 20.82 ± 2.79 years old. The recruitment process is shown in Figure 1.

All participants received an oral explanation of the study’s purpose and signed an informed consent form. The participation was strictly confidential and voluntary. The protocol for this study was approved by the Ethics Committee of the Universidad Politécnica de Madrid (FDRED00000-DML-DATOS-20230609).

### 2.2. Demographic Data

A questionnaire based on previous similar studies was used to record demographic information, including age, height, competition age category, competition weight category, approximate current weight and age at the start of training, years of experience competing, hours of training per week, placement in Spanish Championships, participation in international competitions, international medals, and educational level.

Regarding the questionnaires, the Eating Attitudes Test (EAT-26), the Bulimic Investigatory Test, the Edinburgh (BITE), the Eating Disorders Inventory-3 (EDI-3), and the Depression, Anxiety, and Stress Scale (DASS-21) were used in this study in their previously validated versions.

#### 2.2.1. Eating Attitudes Test (EAT-26)

The EAT-26 questionnaire [27] was used in this study. The EAT-26 has four response options ranging from 0 to 3 (3 = always, 2 = almost always, 1 = often, and 0 = rarely, almost never, or never). These items were divided into three subscales, each corresponding to a facet of eating behavior: (a) dieting, which refers to a pathological refusal to consume high-calorie foods and a preoccupation with physical appearance; (b) bulimia and attention to food, which refers to episodes of binge eating followed by purging behaviors for the purpose of weight loss or weight control; and (c) oral self-control, which reflects self-control with respect to food and assesses the environmental and social forces that stimulate food intake. The total score of the EAT-26 was the sum of the 26 items. Scores above 20 points indicated an eating disorder risk (EAT+). In addition, the SCOFF questionnaire (answering a single question in the affirmative is sufficient to include the participant in the risk group of EDs) was used.

#### 2.2.2. Bulimic Investigatory Test, Edinburgh (BITE)

The BITE was used to identify subjects with binge eating and compensatory behaviors, providing information on cognitive and behavioral aspects of bulimia nervosa [28]. The scores are classified into two subscales: symptoms and severity. On the symptom scale, a score of 20 or more was indicative of bulimia nervosa; 10 to 19 suggests an unusual eating pattern, and less than 10 was within normal limits. On the severity scale, a score of 5 or more was considered clinically significant, and 10 or more indicates a high degree of severity.

#### 2.2.3. Eating Disorders Inventory-3 (EDI-3)

The EDI-3 [29], which is composed of 91 items distributed into 12 main scales, was used for the assessment of EDs. The following were the specific risk scales for the manifestation of eating pathology: thinness obsession (an obsession with having a thin body, a preoccupation with eating, and an intense fear of gaining weight), bulimia (the presence of binge eating and/or compensatory behaviors), and body dissatisfaction (dissatisfaction with the general shape of the body, as well as the rejection of specific areas). The rest of the scales were general psychological scales linked to EDs: low self-esteem (negative self-perception), personal alienation (feelings of emotional emptiness, loneliness, lack of control, and incomprehension), interpersonal insecurity (apprehension about manifesting one’s feelings and thoughts), interpersonal mistrust (feelings of detachment and a propensity to feel trapped in relationships), interoceptive deficits (difficulty interpreting and responding to one’s own and others’ emotional states), emotional maladjustment (emotional instability, impulsivity, and self-destructive behaviors), asceticism (obsessive behaviors of self-discipline, restraint, and self-sacrifice), perfectionism (self or external demand to achieve excessively high goals and objectives), and fear of maturity (insecurity about maturity and a desire to return to childhood). The questionnaire items were answered on a 6-point Likert-type scale (from 1 = never to 6 = always), and the purpose of the questionnaire was not to provide an ED diagnosis but to provide a measure of psychological traits and symptoms common to EDs.

#### 2.2.4. Depression, Anxiety, and Stress Scale (DASS-21)

The DASS-21 was developed to assess markers of distress, including stress, anxiety, and depression [30]. For this study, the Spanish version of the scale was used [31], in which the total number of items was reduced to 21 (7 items per factor: depression, anxiety, and stress). The response scale for all items ranged from 0 (does not apply to me) to 3 (applies to me most of the time). 

### 2.3. Data Collection

Data collection was carried out during a national training camp at the Centro Gallego de Tecnificación Deportiva in Pontevedra, Spain, in October 2022, using a structured, self-administered instrument with questions on demographic and socioeconomic data. Each day of the week before the first training, only one questionnaire (EAT-26, EDI-3, BITE, or DASS-21) was administered with a stipulated time to complete it. All measurement instruments were presented in booklet form. 

### 2.4. Data Analysis

Participants’ data was described using (*M*) and (*SD*). The normality of distribution was tested using the Shapiro–Wilk test, and the homogeneity of variances was tested using Levene’s test, both (*p* > 0.05). Participants were divided into two equal halves using the median, and a Student’s *t*-test was used to determine the differences in EDs based on age, weight, years of training, and competition experience. The effect size was reported using Cohen’s d (small effect [*d* = 0.2–0.5], medium effect [*d* = 0.5–0.8], and large effect [*d* > 0.8]). Cronbach’s Alpha coefficient was calculated to evaluate the reliability of each questionnaire scale and subscales. Finally, linear regression was performed to determine which variables predict the occurrence of EDs. Statistical tests were conducted using IBM SPSS Statistics version 29 for Windows, and the level of significance was set at *p* < 0.05.

## 3. Results

### 3.1. Consistency

The reliability of all questionnaires and their subscales was tested. Cronbach’s Alpha coefficient values are shown in Table 1.

### 3.2. Wrestlers’ Behaviors

The results showed that, on average, the participant’s level of depression (*M* = 4.82 ± 4.22) did not reach the cut-off point established by the DASS-21 for mild depression (*M* > 5). The participants’ level of anxiety (*M* = 4.86 ± 3.81) met the DASS-21 cut-off point for mild anxiety (*M* > 4). The participants’ stress level (*M* = 8.23 ± 4.41) reached the cut-off point marked by the DASS-21 for mild stress (*M* > 8). The frequency distribution of the wrestlers’ behavior is shown in Table 2.

Regarding the BITE questionnaire, the descriptive data of the symptoms scale were (*M* = 7.67 ± 3.39). Scores of 5–10 were not considered an ED, although they were above what is considered normal. On the other hand, the scores in terms of severity were (*M* = 12.36 ± 6.45), with values between 10–20 being considered abnormal. In addition, the frequency distribution shows that 50% of the wrestlers have fasted a whole day, 40% of the wrestlers have used diuretics, 33.3% have used laxatives, 10% have made themselves vomit to lose weight, and 50% of the wrestlers binge eat when they are alone, with 10% of them doing this behavior at least two or three times per week. 

In addition, the EAT-26 showed values of dieting (*M* = 12.88 ± 6.73), bulimia (*M* = 6.13 ± 2.70), and oral control (*M* = 3.50 ± 1.51), with 40.91% of the total participants having a possible ED risk (EAT+). Lastly, the EDI-3 mean descriptive data were driven for thinness (*M* = 18.14 ± 10.77), bulimia (*M* = 12.41 ± 7.04), and body dissatisfaction (*M* = 18.73 ± 12.49), with values over 20 points at frequencies of 46%, 22%, and 33.8%, respectively.

### 3.3. Differences between the Groups—Group Comparison

Differences and effect sizes between the two groups according to the wrestlers’ age, competition experience, training experience, and weight were analyzed. No significant differences were found between the groups of younger and older wrestlers (*p* > 0.05) (Table 3). On the other hand, there were significant differences between wrestlers with greater and lesser years of competitive experience (Table 4). Those with more experience scored higher values in dieting (*p* = 0.038; *d* = 0.95), oral control (*p* = 0.040; *d* = 0.95), asceticism (*p* = 0.018; *d* = 1.10), and maturity fears (*p* = 0.020; *d* = 1.08). Additionally, significant differences were observed between wrestlers with different levels of training experience (Table 5). Thus, athletes with more training experience reported higher levels of bulimia (*p* = 0.028; *d* = 1.01) and over-control (*p* = 0.047; *d* = 0.90). Lastly, there were no significant differences among heavier and lighter wrestlers (*p* > 0.05) (Table 6).

### 3.4. Predictors of Eating Disorders—Regression Analysis

A multiple linear regression was performed to estimate the wrestlers’ factors predicting EDs. The competition experience significantly predicted bulimia severity (*F*[1, 7] = 8.197, *p* = 0.024, and adjusted *R*^2^ = 0.474). Moreover, training experience showed a significant relationship with bulimia symptoms (*F*[1, 20] = 5.449, *p* = 0.030, and adjusted *R*^2^ = 0.175) and bulimia (*F*[1, 20] = 7.432, *p* = 0.013, and adjusted *R*^2^ = 0.234). Similarly, training experience also predicted psychological factors related to EDs, accounting for between 14 and 24% of their variance: personal alienation (*F*[1, 20] = 4.760, *p* = 0.041, and adjusted *R*^2^ = 0.152), emotional dysregulation (*F*[1, 20] = 6.502, *p* = 0.019, and adjusted *R*^2^ = 0.208), asceticism (*F*[1, 20] = 7.517, *p* = 0.013, and adjusted *R*^2^ = 0.237), affective problems (*F*[1, 20] = 5.779, *p* = 0.026, and adjusted *R*^2^ = 0.185), and the DASS total score (*F*[1, 20] = 4.750, *p* = 0.041, and adjusted *R*^2^ = 0.152). The rest of the variables analyzed were not significant predictors (*p* > 0.05).

## 4. Discussion

The main objective of this study was to determine the relationship between different variables of female, elite wrestlers (weight, age, and experience, among others) and the frequencies in which EDs occur in this population. In addition, it sought to establish which factors could be predictors of EDs. In this line, EDs are a serious concern in Olympic wrestling and other weight-dependent sports, with wrestlers resorting to extreme and unhealthy weight-control practices. Pressure to maintain low weights, often influenced by coaches, peers, and the desire for athletic success, results in harmful practices such as severe dietary restrictions and dehydration. These practices lead to negative physical and mental health consequences, affecting athletic performance and overall well-being. In addition, competition and training experience seem to be the most critical factors in the development of EDs in female wrestlers; therefore, it seems essential that athletes of all ages and experience levels receive adequate education on nutrition, healthy weight management, and the promotion of positive body image in order to prevent and address EDs in combat sports [11].

While the mean depression score among our female wrestlers did not surpass the threshold for the mild category according to the DASS-21 questionnaire, 50% exhibit depressive symptoms, and 23% experience moderate to extremely severe depression. This data could be interpreted as a comorbidity factor for certain female wrestlers [32]. In contrast, the anxiety and stress reported by the wrestlers reached the mild threshold according to the DASS-21, with most of the sample exceeding this threshold and a high percentage experiencing severe to extreme anxiety levels (22.7%). In consequence, these three factors could be relevant, as they may influence the onset of EDs and clarify why athletes are at a heightened risk for such conditions [33]. In this line, the prevalence of EDs is higher among athletes than in the general population, especially in female athletes compared to male athletes. Moreover, it is also more common in combat sports that emphasize leanness and weight dependency, as opposed to other sports [34].

Regarding the EDI-3 questionnaire, scores above 20 indicate a risk of developing EDs [27]. Our results showed that, on average, the studied female wrestlers did not reach this threshold of clinical EDs but were close (drive for thinness, M = 18.14 and body dissatisfaction, M = 18.73). It is important to note that our values were clearly higher than those of a similar age and gender in the general population [34]. Additionally, a significant proportion of the participants in our study (drive for thinness, 46%; bulimia, 22%; and body dissatisfaction, 33.8%) are at risk of developing EDs. Thus, our findings were more in line with other combat sports, such as judo, which share the characteristics of being combat-oriented and weight-class-regulated [12].

On the other hand, in the BITE questionnaire, a similar pattern emerged. Elevated symptoms were observed on the assessment scale and were consistent with the study of Oliveira [35], who also found abnormally high scores related to unhealthy eating patterns and body-weight concerns in a sample of young, female athletes participating in various sports, some of them being combat sports. This suggests that female wrestlers exhibit a preoccupation and disturbance in their relationship with food, even when the entire sample did not reach the threshold for a clinical diagnosis of EDs. This phenomenon may be linked to the demands of making weight for competitions [36]. The BITE questionnaire could shed light on why EDs are not detected in female wrestlers despite the presence of symptoms and their severity. In addition, this phenomenon can be attributed to training and competition experience, as the more they compete, the more chances they have to reach a specific weight [11].

Furthermore, our results could be linked to how frequently wrestlers resort to unhealthy methods to quickly achieve their weight goals. Specifically, 40% of the participants admitted to using natural diuretics for weight reduction. These results align with the findings of Gullón-López et al. [37], who highlighted that combat sports athletes often employ dehydration as a strategy to meet weight requirements. Moreover, approximately half of the wrestlers in our study revealed practicing fasting for a full day as a weight-loss strategy. Furthermore, 33.3% acknowledged the use of laxatives for the same purpose, and 10% admitted to inducing vomiting to reduce their body weight. Previous studies, such as the one conducted by Rueda et al. [11], indicated that some wrestlers had employed risky methods prior to weigh-ins. Additionally, it was found that half of the wrestlers admitted to experiencing binge-eating episodes when alone, with 10% of them reporting these episodes occurring at least two or three times per week. Studies like those by van Niekerk et al. [38] and Kiefer [39] suggested that these behaviors appeared to be more common in women than in men. Consequently, all of these behavioral patterns could be predictors of future EDs [5].

Regarding group comparisons, it was observed that more experienced wrestlers presented a higher number of predictive factors, possibly due to their increased participation in competitions, which necessitates more frequent weight adjustments [40]. Furthermore, this could be related to the gradual acquisition of unhealthy behaviors to achieve precise weight goals. In contrast to the study by Rouveix et al. [12] with male judo athletes, where beginners resorted to risky methods for weight loss, our results showed that more experienced female wrestlers exhibit a higher propensity to employ hazardous techniques to reduce their body weight [11]. This disparity may be attributed to differences in weigh-in procedures between judo and wrestling. Thus, in judo, official weigh-ins occur the evening before the competition, while in wrestling, they take place a mere two hours prior to the bout, limiting the wrestlers’ capacity for recovery. Hence, the timing of weigh-ins could be a determining factor. Additionally, contrary to the study by Nishimaki et al. [4], which found that Japanese female wrestlers in lower weight categories tend to be more overweight compared to those in higher weight categories, which could explain the presence of unhealthy eating behaviors, we did not find any effects of weight category on EDs. This discrepancy could be due to limitations in our sample or the timing of the season in which the data were collected.

Experience in training and competition positively explained 18–47% of the variability in the severity scale of bulimia symptoms on the BITE questionnaire. These results are in line with the findings of Thompson and Sherman [23], suggesting that many less experienced athletes share similar risk factors to their older counterparts, although they may be less aware of them. These athletes face an “evolutionary” risk factor, as they are in a life stage characterized by a high risk of developing an ED, either when starting their involvement in a sport or taking their sports career seriously. It is important to note that the majority of athletes with EDs did not initiate their problematic eating patterns in adulthood but rather during adolescence [41]. Similarly, Rueda et al. [11] suggest that more experienced female wrestlers face greater pressure to achieve results, as they have to compete more frequently and, consequently, make weight for competition on several occasions, potentially leading to additional pressure. Thus, wrestlers with EDs may feel less fearful about maintaining the disorder rather than trying to overcome it. This fact may be due to different motivating factors, depending on the disorder cycle time [23]. In addition, the desire to achieve optimal health and performance in sports appears to be one of the most important factors for athletes to recover themselves after EDs [42].

Although our study provides valuable and relatively unexplored insights, there are certain limitations that need to be mentioned. First, the sample participants exclusively consisted of members from the national women’s wrestling team, raising the question of whether these issues are gender-specific or also apply to male athletes. Another limitation is the temporal nature of the data collection, as it represents a specific point in the season—the preseason—when the weight requirements could be lesser than in competition season periods. Additionally, only some age categories were analyzed. This fact could hide information about how younger wrestlers could be affected by EDs. Consequently, future research should investigate whether the findings of our study are consistent across gender, other weight-category sports, and weight categories. Lastly, the implementation of a longitudinal study would provide a more in-depth understanding of how EDs are developed over time.

## 5. Conclusions

This study suggests that differences in EDs were found between groups with higher and lower training and competitive experience but not in age and weight categories. Therefore, competitions and training experiences appear to be the most critical variables related to EDs and psychological problems. This implies that competition intensity and training time play a crucial role in the mental health of elite female athletes. Additionally, although the group of wrestlers does not meet the threshold for EDs according to the questionnaire’s standard values, there is a high frequency of behaviors associated with EDs; therefore, many athletes are at a high risk of developing EDs during their athletic careers. Lastly, a high percentage of wrestlers experience high levels of anxiety, depression, and stress, which could lead to the development of EDs.

## 6. Practical Application

To address this issue effectively, a collaborative approach involving coaches, health professionals, and athletes is paramount. Coaches and support staff must undergo comprehensive training to adeptly identify signs that are indicative of EDs, offer necessary support, and seamlessly facilitate referrals to specialized healthcare professionals when warranted. Simultaneously, athletes require thorough education on the risks associated with EDs, coupled with relentless encouragement to prioritize their overall health and well-being over the pursuit of extreme weight-control measures. Consequently, it is imperative for sports organizations and Olympic committees to establish robust policies that champion healthy weight-management practices. These policies should encompass provisions for ample resources and dedicated support systems, thus fortifying athletes’ ability to maintain peak health while engaging in elite-level competition. Through the diligent implementation of these recommendations, stakeholders can collaboratively cultivate an environment that steadfastly upholds the health and well-being of athletes engaged in Olympic wrestling and other combat sports.

## Figures and Tables

**Table 1:** Results for each scale and subscale with their level of internal consistency.

Factor	*n*	Items	Min	Max	M	SD	Cronbach’s Alpha
BULIMIA							
Symptoms	22	30	3.00	23.00	12.05	6.09	0.883
DASS	22	21	4.00	47.00	17.91	10.56	0.892
Depression	22	7	0.00	14.00	4.82	4.22	0.846
Anxiety	22	7	0.00	15.00	4.86	3.81	0.734
Stress	22	7	2.00	18.00	8.23	4.41	0.776
EAT-26	22	26	11.00	36.00	22.50	8.42	0.912
Dieting	22	13	4.00	23.00	12.88	6.73	0.920
Bulimia	22	6	3.00	9.00	6.13	2.70	0.556
Oral control	22	7	2.00	6.00	3.50	1.51	0.516
EDI-3							
Eating Disorder Risk Scales							
Drive for thinness	22	7	2.00	34.00	18.14	10.77	0.948
Bulimia	22	8	0.00	25.00	12.41	7.04	0.861
Body dissatisfaction	22	10	2.00	47.00	18.73	12.49	0.900
Psychological scales							
Low self-esteem	22	6	0.00	26.00	10.00	7.65	0.939
Personal alienation	22	7	1.00	32.00	10.77	7.23	0.861
Interpersonal insecurity	22	7	5.00	22.00	14.82	4.87	0.480
Interpersonal alienation	22	7	7.00	23.00	12.45	4.34	0.561
Interoceptive deficits	22	9	5.00	34.00	16.23	8.56	0.853
Emotional dysregulation	22	8	2.00	30.00	11.55	6.22	0.773
Perfectionism	22	6	7.00	28.00	17.27	6.16	0.653
Asceticism	22	7	1.00	25.00	12.14	5.88	0.619
Maturity fears	22	8	0.00	32.00	18.18	7.72	0.800
Composites							
EDRC	22	25	10.00	95.00	49.27	25.52	0.940
IC	22	13	1.00	58.00	20.77	14.33	0.944
IPC	22	14	14.00	41.00	27.27	8.10	0.688
APC	22	17	7.00	64.00	27.77	13.70	0.891
OC	22	13	17.00	48.00	30.32	8.81	0.727
GMPC	22	91	76.00	248.00	161.14	51.92	0.947

Note. EDRC: Eating Disorder Risk Composite; IC: Ineffectiveness Composite; IPC: Interpersonal Problems Composite; APC: Affective Problems Composite; OC: Over-control Composite; GPMC: General Psychological Maladjustment Composite.

**Table 2:** Frequency distribution of the depression, anxiety, and stress categories.

	Depression		Anxiety		Stress	
	*n*	%	*n*	%	*n*	%
No symptoms	11	50	9	40.9	10	45.5
Mild	6	27.3	5	22.7	3	13.7
Moderate	2	9.1	3	13.7	6	27.3
Severe	2	9.1	1	4.5	2	9.1
Extremely severe	1	4.5	4	18.2	1	4.5
TOTALS	22	100	22	100	22	100

Notes: *n* = number of participants, % = percentage of affected wrestlers.

**Table 3:** Results according to the age of the wrestlers (median = 21).

Factor	Older ^a^		Younger ^b^				95% CI		
	M	SD	M	SD	t(20)	*p*	LL	UL	d
BULIMIA									
Symptoms	13.27	6.44	10.82	5.76	0.94	0.357	−2.98	7.89	0.40
DASS	19.82	12.42	16.00	8.46	0.84	0.410	−5.64	13.27	0.36
Depression	6.09	4.41	3.55	3.78	1.45	0.162	−1.11	6.20	0.62
Anxiety	4.18	4.40	5.55	3.17	−0.83	0.414	−4.78	2.05	−0.36
Stress	9.55	5.43	6.91	2.74	1.44	0.171	−1.28	6.55	0.61
EAT-26	23.27	14.70	18.09	11.63	0.92	0.370	−6.61	16.97	0.39
Dieting	13.73	10.15	10.27	7.84	0.89	0.382	−4.61	11.52	0.38
Bulimia	5.82	3.46	4.82	2.52	0.77	0.448	−1.69	3.69	0.33
Oral control	3.73	3.04	3.00	3.69	0.50	0.619	−2.28	3.73	0.22
EDI-3									
Eating Disorder Risk Scales									
Drive for thinness	19.27	9.94	17.00	11.91	0.49	0.632	−7.48	12.03	0.21
Bulimia	12.91	5.79	11.91	8.37	0.33	0.748	−5.40	7.40	0.14
Body dissatisfaction	16.64	12.70	20.82	12.53	−0.78	0.446	−15.40	7.04	−0.33
Psychological scales									
Low self-esteem	9.91	8.07	10.09	7.61	−0.05	0.957	−7.16	6.79	−0.02
Personal alienation	11.36	8.23	10.18	6.43	0.38	0.711	−5.39	7.75	0.16
Interpersonal insecurity	13.73	3.07	15.91	6.14	−1.05	0.309	−6.60	2.24	−0.45
Interpersonal alienation	11.91	5.05	13.00	3.66	−0.58	0.568	−5.01	2.83	−0.25
Interoceptive deficits	15.00	8.53	17.45	8.82	−0.66	0.515	−10.17	5.27	−0.28
Emotional dysregulation	11.64	7.15	11.45	5.48	0.07	0.947	−5.48	5.85	0.03
Perfectionism	18.00	7.32	16.55	4.99	0.54	0.592	−4.12	7.03	0.23
Asceticism	13.64	6.14	10.64	5.48	1.21	0.241	−2.18	8.18	0.52
Maturity fears	15.73	7.96	20.64	6.96	−1.54	0.139	−11.56	1.74	−0.66
Composites									
EDRC	48.82	24.12	49.73	28.03	−0.08	0.936	−24.17	22.35	−0.04
IC	21.27	15.68	20.27	13.59	0.16	0.875	−12.05	14.05	0.07
IPC	25.64	7.27	28.91	8.89	−0.94	0.356	−10.50	3.95	−0.40
APC	26.64	14.78	28.91	13.15	−0.38	0.707	−14.71	10.17	−0.16
OC	29.36	6.56	31.27	10.85	−0.50	0.624	−10.00	6.18	−0.21
GMPC	158.09	44.58	164.18	60.45	−0.27	0.791	−53.33	41.15	−0.12

^a^ *n* = 11. ^b^ *n* = 11. CI = confidence interval; LL = lower limit; UL = upper limit. EDRC: Eating Disorder Risk Composite; IC: Ineffectiveness Composite; IPC: Interpersonal Problems Composite; APC: Affective Problems Composite; OC: Over-control Composite; GPMC: General Psychological Maladjustment Composite.

**Table 4:** Results according to the competition experience of the wrestlers (median = 7.00).

Factor	More Experience ^a^		Less Experience ^b^				95% CI		
	M	SD	M	SD	t(20)	*p*	LL	UL	d
BULIMIA									
Symptoms	14.17	5.97	9.50	5.45	1.90	0.072	−0.46	9.80	0.81
DASS	20.08	12.81	15.30	6.75	1.06	0.301	−4.62	14.18	0.46
Depression	6.08	4.93	3.30	2.67	1.60	0.126	−0.85	6.42	0.68
Anxiety	4.92	4.56	4.80	2.90	0.07	0.945	−3.37	3.60	0.30
Stress	9.08	5.30	7.20	2.97	1.05	0.308	−1.89	5.66	0.43
EAT-26	25.67	14.19	14.70	9.38	2.09	0.050 *	0.02	21.92	0.89
Dieting	15.58	9.56	7.70	6.38	2.22	0.038 *	0.49	15.28	0.95
Bulimia	5.42	3.03	5.20	3.12	0.16	0.871	−2.53	2.96	0.07
Oral control	4.67	3.77	1.80	1.81	2.19	0.040 *	0.14	5.59	0.94
EDI-3									
Eating Disorder Risk Scales									
Drive for thinness	22.08	10.37	13.40	9.66	2.02	0.057	−0.30	17.67	0.86
Bulimia	14.00	5.88	10.50	8.13	1.17	0.255	−2.73	9.73	0.50
Body dissatisfaction	22.67	14.75	14.00	7.26	1.79	0.091	−1.56	18.89	0.72
Psychological scales									
Low self-esteem	12.00	8.51	7.60	6.04	1.37	0.186	−2.30	11.10	0.59
Personal alienation	12.58	8.16	8.60	5.56	1.31	0.206	−2.37	10.33	0.56
Interpersonal insecurity	15.75	4.11	13.70	5.66	0.98	0.337	−2.30	6.40	0.42
Interpersonal alienation	13.25	4.97	11.50	3.44	0.94	0.359	−2.14	5.64	0.40
Interoceptive deficits	17.17	8.61	15.10	8.82	0.55	0.586	−5.71	9.84	0.24
Emotional dysregulation	12.42	6.89	10.50	5.46	0.71	0.485	−3.70	7.54	0.30
Perfectionism	16.92	6.57	17.70	5.95	−0.29	0.774	−6.41	4.84	-0.12
Asceticism	14.75	6.05	9.00	4.00	2.57	0.018 *	1.08	10.42	1.10
Maturity fears	17.58	8.83	18.90	6.54	−0.39	0.700	−8.35	5.72	-0.17
Composites									
EDRC	58.75	26.39	37.90	20.12	2.05	0.054	−0.38	42.08	0.88
IC	24.58	15.96	16.20	11.19	1.40	0.178	−4.14	20.90	0.60
IPC	29.00	8.10	25.20	8.01	1.10	0.284	−3.40	11.00	0.47
APC	29.58	14.24	25.60	13.43	0.67	0.510	−8.42	16.38	0.29
OC	32.33	8.62	27.90	8.85	1.19	0.249	−3.36	12.22	0.51
GMPC	178.75	49.97	140.00	48.25	1.84	0.081	−5.20	82.70	0.79

^a^ *n* = 11. ^b^ *n* = 11. CI = confidence interval; LL = lower limit; UL = upper limit. EDRC: Eating Disorder Risk Composite; IC: Ineffectiveness Composite; IPC: Interpersonal Problems Composite; APC: Affective Problems Composite; OC: Over-control Composite; GPMC: General Psychological Maladjustment Composite. * *p* < 0.05.

**Table 5:** Results according to the training experience of the wrestlers (median = 9.50).

Factor	More Experience ^a^		Less Experience ^b^				95% CI		
	M	SD	M	SD	t(20)	*p*	LL	UL	d
BULIMIA									
Symptoms	13.91	6.20	10.18	5.64	1.47	0.156	−1.54	9.00	0.63
DASS	19.09	12.00	16.73	9.32	0.52	0.612	−7.19	11.92	0.22
Depression	5.27	3.80	4.36	4.74	0.50	0.625	−2.91	4.73	0.21
Anxiety	4.55	4.37	5.18	3.34	−0.38	0.705	−4.09	2.82	-0.16
Stress	9.27	5.33	7.18	3.16	1.12	0.276	−1.81	5.99	0.48
EAT-26	24.18	13.11	17.18	12.94	1.26	0.222	−4.59	18.59	0.54
Dieting	14.36	9.52	9.64	8.25	1.24	0.228	−3.20	12.65	0.53
Bulimia	6.18	2.89	4.45	2.98	1.38	0.183	−0.88	4.34	0.59
Oral control	3.64	3.04	3.09	3.70	0.38	0.710	−2.47	3.56	0.16
EDI-3									
Eating Disorder Risk Scales									
Drive for thinness	20.55	10.19	15.73	11.26	1.05	0.305	−4.73	14.37	0.45
Bulimia	15.64	6.31	9.18	6.43	2.38	0.028 *	0.79	12.12	1.01
Body dissatisfaction	19.73	13.56	17.73	11.91	0.37	0.717	−9.35	13.35	0.16
Psychological scales									
Low self-esteem	12.27	8.34	7.73	6.48	1.43	0.169	−2.10	11.19	0.61
Personal slienation	13.45	8.47	8.09	4.72	1.84	0.081	−0.73	11.46	0.78
Interpersonal insecurity	15.55	4.11	14.09	5.63	0.69	0.497	−2.93	5.84	0.30
Interpersonal alienation	12.82	5.06	12.09	3.70	0.38	0.704	−3.21	4.67	0.16
Interoceptive deficits	18.82	9.23	13.64	7.35	1.46	0.161	−2.24	12.60	0.62
Emotional dysregulation	13.00	7.18	10.09	4.99	1.10	0.283	−2.59	8.41	0.47
Perfectionism	17.82	7.03	16.73	5.44	0.41	0.688	−4.50	6.68	0.17
Asceticism	14.27	6.36	10.00	4.71	1.79	0.088	−0.70	9.25	0.76
Maturity fears	19.73	8.33	16.64	7.10	0.94	0.360	−3.80	9.98	0.40
Composites									
EDRC	55.91	25.34	42.64	25.08	1.24	0.231	−9.15	35.69	0.53
IC	25.73	16.35	15.82	10.50	1.69	0.106	−2.31	22.13	0.72
IPC	28.36	8.39	26.18	8.05	0.62	0.541	−5.13	9.50	0.27
APC	31.82	15.05	23.73	11.48	1.42	0.172	−3.81	19.99	0.60
OC	34.00	7.33	26.64	8.90	2.12	0.047 *	0.11	14.62	0.90
GMPC	180.64	49.88	141.64	48.34	1.86	0.077	−4.69	82.69	0.79

^a^ *n* = 11. ^b^ *n* = 11. CI = confidence interval; LL = lower limit; UL = upper limit. EDRC: Eating Disorder Risk Composite; IC: Ineffectiveness Composite; IPC: Interpersonal Problems Composite; APC: Affective Problems Composite; OC: Over-control Composite; GPMC: General Psychological Maladjustment Composite. * *p* < 0.05.

**Table 6:** Results according to the weight of the wrestlers (median = 63.75).

Factor	Heaviest ^a^		Lightest ^b^				95% CI		
	M	SD	M	SD	t(20)	*p*	LL	UL	d
BULIMIA									
Symptoms	11.36	6.64	12.73	5.73	−0.52	0.612	−6.88	4.15	−0.22
DASS	13.73	8.20	22.09	11.33	−1.98	0.061	−17.16	0.43	−0.85
Depression	3.27	3.04	6.36	4.78	−1.81	0.085	−6.65	0.47	−0.77
Anxiety	3.64	3.20	6.09	4.11	−1.56	0.134	−5.73	0.82	−0.67
Stress	6.82	3.97	9.64	4.54	−1.55	0.137	−6.61	0.98	−0.66
EAT-26	21.55	13.93	19.82	10.64	0.30	0.767	−10.28	13.74	0.13
Dieting	13.36	10.19	10.64	5.82	0.70	0.492	−5.40	10.85	0.30
Bulimia	4.82	2.23	5.82	3.36	−0.77	0.448	−3.69	1.69	−0.33
Oral control	3.36	3.04	3.36	5.16	0.00	0.999	−3.02	3.02	0.00
EDI-3									
Eating Disorder Risk Scales									
Drive for thinness	19.09	12.30	17.18	9.50	0.41	0.688	−7.86	11.68	0.17
Bulimia	12.45	6.73	12.36	7.67	0.03	0.977	−6.33	6.51	0.01
Body dissatisfaction	23.09	13.38	14.36	10.35	1.71	0.102	−1.91	19.36	0.73
Psychological scales									
Low self-esteem	8.09	6.56	11.91	8.48	−1.18	0.251	−10.56	2.93	−0.50
Personal slienation	9.27	5.41	12.27	8.70	−0.97	0.343	−9.44	3.44	−0.41
Interpersonal insecurity	14.00	4.63	15.64	5.18	−0.78	0.444	−6.01	2.73	−0.33
Interpersonal alienation	11.91	3.18	13.00	5.37	−0.58	0.568	−5.01	2.83	−0.25
Interoceptive deficits	14.73	7.86	17.73	9.34	−0.82	0.425	−10.68	4.68	−0.35
Emotional dysregulation	10.27	4.84	12.82	7.36	−0.96	0.349	−8.09	2.99	−0.41
Perfectionism	17.00	5.62	17.55	6.92	−0.20	0.841	−6.15	5.06	−0.09
Asceticism	11.64	5.80	12.64	6.20	−0.39	0.700	−6.34	4.34	−0.17
Maturity fears	21.18	7.03	15.18	7.48	1.94	0.067	−0.45	12.45	0.83
Composites									
EDRC	54.64	29.70	43.91	20.55	0.99	0.336	−11.99	33.44	0.42
IC	17.36	11.40	24.18	16.61	−1.12	0.275	−19.49	5.85	−0.48
IPC	25.91	7.02	28.64	9.19	−0.78	0.443	−10.00	4.55	−0.33
APC	25.00	11.76	30.55	15.46	−0.95	0.355	−17.76	6.67	−0.40
OC	32.82	8.85	27.82	8.41	1.36	0.190	−2.68	12.68	0.58
GPMC	162.45	53.91	159.82	52.45	0.12	0.909	−44.67	49.94	0.05

Note. ^a^ *n* = 11. ^b^ *n* = 11. CI = confidence interval; LL = lower limit; UL = upper limit. EDRC: Eating Disorder Risk Composite; IC: Ineffectiveness Composite; IPC: Interpersonal Problems Composite; APC: Affective Problems Composite; OC: Over-control Composite; GPMC: General Psychological Maladjustment Composite.

## Data Availability

The data presented in this study are available on request from the corresponding author. The data are not publicly available due to privacy.
